# Co-creating the future: participatory cities and digital governance

**DOI:** 10.1098/rsta.2024.0113

**Published:** 2024-11-13

**Authors:** Dirk Helbing, Sachit Mahajan, Dino Carpentras, Monica Menendez, Evangelos Pournaras, Stefan Thurner, Trivik Verma, Elsa Arcaute, Michael Batty, Luis M. A. Bettencourt

**Affiliations:** ^1^ Computational Social Science, ETH Zurich, Zürich, Switzerland; ^2^ Complexity Science Hub Vienna, Vienna, Austria; ^3^ NYU Abu Dhabi, Abu Dhabi, UAE; ^4^ School of Computing, University of Leeds, Leeds, UK; ^5^ Bristol Digital Futures Institute and School for Policy Studies, University of Bristol, Bristol, UK; ^6^ Centre of Advanced Spatial Analysis, University College London, London, UK; ^7^ University of Chicago, Chicago, IL, USA

**Keywords:** participatory cities, socio-technical system, digital governance

## Abstract

The digital revolution, fuelled by advancements in social media, Big Data, the Internet of Things and Artificial Intelligence, is reshaping our urban landscapes into ‘participatory cities’. These cities leverage digital technologies to foster citizen engagement, collaborative decision-making and community-driven urban development, thus unlocking new potentials while confronting emerging threats. Such technologies are empowering individuals and organizations in ways that were unimaginable just a few years ago. They do, however, introduce new risks and vulnerabilities that must be carefully managed. Hence, socio-technical innovation is urgently needed. In this connection, open-source technologies, participatory approaches and new forms of governance are becoming more popular and relevant. This theme issue looks into the tangible impacts of these technological advancements, with a focus on participatory cities. It aims to explain how digital tools are used in cities to tackle urban challenges, improve governance and promote sustainability. Through a collection of in-depth analyses, case studies and real-world examples, this issue seeks to offer a comprehensive understanding of the digital governance frameworks underpinning participatory cities. By offering a platform for multidisciplinary discourse, this theme issue endeavours to contribute to the broader narrative of shaping a more resilient, sustainable and democratic urban future in the digital age.

This article is part of the theme issue ‘Co-creating the future: participatory cities and digital governance’.

## 1. Introduction

On 11 September 2023, everything should start changing for the better forever. This is what we hoped when we were having a workshop on Co-creating the future at the Complexity Science Hub, Vienna, from the 11th to the 13th of September, 2023.^1^ The workshop aimed at a fundamental paradigm change: Cities should not be run like businesses and not like machines [1]—also in the age of Big Data and AI [2].

This, in part, is questioning the way ‘Smart Cities’ were traditionally conceived. Using the Internet of Things, millions of sensors would collect Big Data to optimize, control and automate our cities—so went the idea. While this may sound great at first, it just did not work. Not only did people often object to the underlying surveillance-based and data-driven approach, as Google had to learn in Toronto, for example [3]. At the Wilton Park event on disrupting cities through Technology’ [4], various stakeholder were raising further issues: neither the mayors of smart cities nor the technology companies running them seemed to be really satisfied with the results.

In fact, as was recently stressed in a paper on the ethics of smart cities [5]: ‘Cities are not just giant optimisation problems, nor are they giant entertainment parks, where pre-manufactured experiences are consumed. Cities are places where people meet, communicate, make friends, and fall in love. In other words, cities are first and foremost about people’. Accordingly, we need ‘participatory cities’, in which we can co-create our neighbourhoods and our future, thereby making the cities, in which we live, ‘*our* cities’. Moreover, Smart Cities should be designed for self-organization, and digital technology can assist this [6].

We chose Vienna as the location for our workshop for good reasons. It is the city that consistently achieves top ratings in the quality-of-life rankings [7,8]—far ahead of the metropoles of the digital technology centres around the world. Remarkably, Vienna had engaged in digitalization early on, but it chose an open, participatory approach (as they say in German: ‘Wien digt anders’ [9]). This approach turned out to be publicly agreeable, while most of the citizens of the supposedly 240 Smart Cities throughout Europe [10] seem to know close to nothing about what is actually done to make their cities smart. This lack of transparency implies democratic deficits, as does the widespread trend to undermine the public agency of people. This can be quite far-reaching. Some Smart City concepts even seem to consider a post-voting and post-decision society.^2^

However, there are (at least) two ways of using digital technologies in societies: one way relies on a surveillance-based, data-driven, AI-controlled approach to automate societies, thereby increasingly replacing governance by cybernetics; the other is assisting bottom-up coordination and self-organization [11] in the spirit of synergetics [12–14]. Accordingly, novel participatory approaches have recently been developed, which range from participatory budgeting (PB) [15,16] over participatory sustainability [17,18] to participatory resilience [19,20].

Therefore, we believe that the digital revolution, fuelled by advancements in social media, Big Data, the Internet of Things (IoT), and Artificial Intelligence (AI), will not only reshape our cities; it will eventually also turn our urban landscapes into ‘participatory cities’. These cities leverage digital technologies to foster citizen engagement, collaborative decision-making and community-driven urban development, thus unlocking new potentials while confronting emerging threats [21].

Such technologies are empowering individuals and organizations in ways that were unimaginable just a few years ago. They do, however, introduce new risks and vulnerabilities that must be carefully managed. Hence, socio-technical innovation is urgently needed. In this connection, open-source technologies, participatory approaches and new forms of governance are becoming more popular and relevant.

This theme issue explores some of the tangible impacts of these socio-technical advancements, with a focus on participatory cities. It aims to explain how digital tools can be used in cities to tackle urban challenges, improve governance and promote sustainability. Through a collection of in-depth analyses, case studies and real-world examples, we seek to offer a better understanding of the digital governance frameworks underpinning participatory cities.

## 2. Overview of papers collected in this theme issue

The papers in this theme issue collectively explore the transformative role of digital technologies in shaping participatory urban processes. From facilitating citizen engagement to challenging conventional governance frameworks of smart cities, these contributions address the critical balance between technology, civic participation and societal values. Together, they illustrate how digital tools can help co-create urban spaces while highlighting the challenges that arise when governance is mediated by technology.

In *Large language models (LLMs) as agents for augmented democracy* [22], the potential of AI to enhance civic participation is explored through the development of personalized digital twins that model citizens’ preferences. By using six off-the-shelf large language models (LLMs), fine-tuned with data from Brazil’s 2022 election experiment, this paper illustrates how AI can bridge gaps in citizen engagement and foster more responsive democratic processes. The study demonstrates that LLM-augmented samples provide more accurate estimates of population-level preferences than non-augmented samples alone, outperforming traditional ‘bundle rule’ predictions based on political orientation. This contribution deepens the discussion on the role of AI in participatory governance, showing how technology can be leveraged to model individual political preferences and engage citizens in a more direct manner, potentially enabling forms of democracy that interpolate between representative and direct democracy.

The paper *Complex systems perspective in assessing risks in artificial intelligence* [23] extends the conversation by examining the long-term risks associated with AI through a complex systems lens. It argues for a holistic approach to AI risk assessment that considers the socio-political and economic feedback loops created by AI technologies. The authors advocate for public participation in the governance of AI systems, emphasizing that AI risks are inherently systemic and cannot be reduced to technical concerns alone. The paper presents a case study of the UK’s school examination algorithm during COVID-19 to illustrate the complex interactions between AI systems and societal inequalities. It proposes specific methods for improving risk assessments, including computational modelling to simulate long-term effects of AI on minority representation and the use of competency groups to incorporate diverse perspectives in the assessment process.

*Artefact design and societal worldview* [24] looks into the power of technological artefacts in shaping societal values and worldviews. By using computational models, the paper demonstrates how the design of artefacts, such as voting advice applications, can influence public opinion and cluster societal views. The authors argue for participatory design processes to mitigate the risks of biased technological artefact development, aligning with the theme of co-creation and inclusivity. This paper underscores the importance of designing digital tools that reflect the values and needs of diverse societal groups, furthering the discourse on participatory urban governance.

*Empowering minorities and everyone in participatory budgeting: an agent-based modelling perspective* [25] explores the integration of collective intelligence (CI) with participatory budgeting (PB). The study examines how the naive integration of CI in PB may disadvantage minority groups, as the quality of solutions would be proportional to the group size. By introducing a common knowledge base, this study demonstrates the possibility of enhancing fairness, enabling minority groups to benefit from the work of the majority and vice versa. Indeed, a common knowledge base allows all groups to produce new solutions that can be used by everyone, independently of their group’s size. This approach leads to better overall solutions while maintaining fairness, promoting collaboration and encouraging shared innovation.

The paper *Computational diplomacy: how ‘hackathons for good’ feed a participatory future for multilateralism in the digital age* [26] provides new perspectives on the subject of computational diplomacy. It studies the role that hackathons can play to build a community of software and hardware developers addressing, for example, the sustainable development goals. Besides short-term innovation, it also engages in the long-term production of public benefits: ‘This bottom-up approach to digital governance integrates software development, human collective intelligence, and collective action, creating a dynamic model for transformative change’.

The paper *Navigating the cohesion-diversity trade-off: understanding the role of facilitators in co-creation using agent-based modelling* [27] provides a foundational look at the internal dynamics of co-creation within participatory cities. Using agent-based modelling (ABM), the author reveals the critical role of facilitators in balancing cohesion and diversity in collaborative environments. The study uncovers what the authors term the ‘Facilitator’s Paradox’, where efforts to enhance group cohesion can inadvertently suppress diversity, reducing the innovative potential of the group. This tension is central to the discourse on participatory cities, where the challenge lies in maintaining a diverse set of perspectives while fostering enough cohesion to ensure actionable governance. The paper situates itself within the broader scope of the theme issue by emphasizing the importance of understanding group dynamics in the context of urban co-creation.

*Addressing the urban congestion challenge based on traffic bottlenecks* [28] explores how to alleviate urban congestion by leveraging real-time data to detect and analyse traffic bottlenecks. By integrating complex systems science with urban planning, the study aims to create a decentralized traffic management framework that enables urban planners to identify bottlenecks early and mitigate their impact on traffic flow. The study highlights the benefits of prioritizing interventions at critical points in the network, offering a scalable and dynamic solution for traffic optimization. The research emphasizes the importance of combining real-time data with urban planning principles, demonstrating how such an approach can enhance both mobility and the liveability of cities by addressing congestion before it becomes a significant problem.

Given that the world is in a rapid urbanization process and cities are, hence, going through a process of re-organization, *Analysing macroscopic traffic rhythms and city size in affluent cities: insights from a global panel data of 25 cities* [29] studies how city size affects traffic capacities and urban traffic management capabilities. The paper reveals new nsights from a global panel data of 25 cities into how physical aspects related to urban form influence mobility patterns and how traffic management policies can become more sustainable. For this, it investigates various cities, primarily situated in Europe, North America and East Asia, considering population sizes, geographical extents and global locations.

*Fair voting outcomes with impact and novelty compromises? Unraveling biases in electing participatory budgeting winners* [30] addresses the complexities and potential biases of PB voting systems. The study explores how voting methods, like equal shares, which aim for fairness and proportional representation, may inadvertently lead to the underrepresentation of high-impact but costly projects, such as infrastructure. Conversely, these methods tend to favour projects in education, welfare and culture, which are lower in cost but potentially impactful in other ways. Using a large-scale analysis of real-world PB elections, the authors introduce a novel framework for assessing both the impact and novelty of elected projects, providing a deeper understanding of how fairness-oriented voting algorithms can affect the outcomes of PB processes. The study highlights the need to balance fairness and impact, particularly for sustainability-focused projects, and discusses ways to mitigate biases in future PB campaigns.

The article *Cities beyond proximity* [31] takes a critical look at the concept of the 15 minute city, which promotes the idea that urban areas function better when essential services are within a short walking or cycling distance. While this model encourages sustainability and convenience, the authors highlight its limitations, arguing that focusing solely on proximity overlooks critical issues like social equity, service quality and local identity. The paper proposes moving towards ‘value-based cities’, which consider not only physical proximity but also factors such as diversity, community participation and the quality of public spaces. The authors stress the importance of combining top-down urban planning with grassroots, bottom-up initiatives to create more inclusive, resilient and democratic urban spaces that cater to diverse needs and enhance local identity and engagement.

*Exploring legitimacy in a municipal budget decision in Switzerland: empirical insights into citizens’ perceptions* [32] investigates how citizens perceive the legitimacy of municipal budget decisions, focusing on input, throughput and output legitimacy. Using data from case studies in Switzerland, the authors analyse whether these dimensions are assessed independently or collectively by citizens. The study highlights how traditional majoritarian voting systems lead to more correlated perceptions of legitimacy, whereas innovative PB processes, such as the Method of Equal Shares, result in separate evaluations of these dimensions. This research provides valuable insights for policymakers seeking to enhance legitimacy in democratic decision-making processes, emphasizing the importance of fairness and participation in shaping citizens’ acceptance of budget outcomes.

*Participatory design based on opinion pooling* [33] explores innovative methods for urban planning and land development by integrating participatory geodesign with opinion pooling techniques. The authors propose a framework that utilizes land suitability mapping, where conflicting factors are combined through hierarchical weighting and Markov averaging. This allows for a collective resolution of differing opinions among stakeholders, enabling the selection of optimal development locations. The study demonstrates how group dynamics and expert opinions can be synthesized to improve public participation in urban design, offering a collaborative approach that balances various stakeholder perspectives.

*Co-designing transport models as a heuristic planning tool* [34] realizes that conventional urban planning is insufficient to deal with the growing degree of the related complexity. Therefore, a new collaborative process is proposed, which integrates design thinking approaches with agent-based transport simulations and ‘requires urban designers and transport modellers to co-create goal-driven and agile transport models that act as a heuristic tool to guide planning decisions in early design stages’. A case study is presented to demonstrate how to implement this approach in order to investigate the expected effects of automated vehicles on urban planning.

In a more application-oriented discussion of participatory technology, *Mapping sidewalk accessibility with smartphone imagery and Visual AI: a participatory approach* [35] presents a novel Sidewalk AI Scanner for mapping sidewalk accessibility. This paper showcases how people can use smartphone technology to contribute to urban planning by capturing video data of sidewalks, which is then analysed using visual AI to assess accessibility features. The study validates the feasibility of this approach, focusing particularly on estimating sidewalk width, and demonstrates promising accuracy results across different urban settings. The Sidewalk AI Scanner aligns with the participatory cities’ ethos, offering a scalable and low-cost solution to address the lack of sidewalk accessibility data. By empowering individuals to directly contribute to urban data collection, this study exemplifies how participatory technologies can make cities more inclusive and equitable, while also highlighting the potential challenges in implementing such systems across diverse urban environments.

A critical perspective is brought forward by *Resisting technological inevitability: Google Wing’s delivery drones and the fight for our skies* [36]. This paper examines the resistance to corporate-driven technological projects in urban environments. The paper provides a critical examination of such technological projects in urban environments, focusing on Google Wing’s drone delivery trials in Canberra, Australia. The authors analyse how narratives of technological inevitability are used by corporations and governments to facilitate the introduction of new technologies and how these narratives are resisted by local communities. The study presents a detailed case study of the ‘Bonython Against Drones’ community group, which successfully challenged Google Wing’s operations and narratives. The paper highlights the importance of community action in contesting top-down technological implementations and reclaiming public spaces, particularly the sky as a commons.

*No one-size-fits-all: Multi-actor perspectives on public participation and digital participatory platforms* [37] investigates the perspectives of different urban actors with respect to public participation. It turns out that no-one-size-fits-all. The paper delivers important insights and guidelines for effective public participation as well as a typology of digital participation platforms to connect diverse needs. ‘The guidelines provide concrete recommendations to support both urban practitioners and interface designers in designing participatory strategies and platforms’.

### 3. Lessons learned

By offering a platform for multidisciplinary discourse, this theme issue contributes to the broader narrative on shaping a more resilient, sustainable and democratic urban future in the digital age. It addresses the intersection of digital technology and society, highlighting the emergent model of participatory cities alongside digital governance. participatory cities embody a citizen-centric urban development paradigm, where digital technologies such as social media, Big Data, IoT and AI obviously play a pivotal role. These technologies are transforming the ways we communicate, learn, work and make decisions, creating new opportunities for individual and collective empowerment. However, these technologies also introduce new vulnerabilities and challenges, including issues related to privacy, security, the digital divide and the ethical implications of AI.^3^ Therefore, an exploration of the impacts and responses to these technologies is timely and necessary.

Innovations in governance, open-source technologies and participatory approaches present promising strategies for managing these challenges and for fostering socio-technical innovation. New developments in these areas are presented and discussed in this theme issue. For example, the subjects of Collective Intelligence and Digital Democracy [38] as well as new forms of urbanism represent emerging trends in leveraging digital technologies for public good and citizen empowerment.

On the scientific and methodological side, the theme issue reflects progress at the intersection of areas such as Computational Social Science, Data Science, Complexity Science and interdisciplinary physics, as well as traffic modelling and urban planning.

The proposed theme issue is particularly timely given the accelerated digitization in response to the COVID-19 pandemic, making digital technologies an even more central part of our lives. It is also in line with recent policy developments, such as the European Union’s Digital Services Act and Artificial Intelligence Act, aimed at regulating digital technologies and protecting user rights.

Moreover, this theme issue aspires to building upon preceding work by connecting diverse fields with each other—from technological innovations to social sciences, urban planning to evidence-based policy approaches. This multidisciplinary lens will facilitate a robust exploration of participatory cities and digital governance, unravelling the intricacies of the digital revolution, while propelling discussions on sustainable, inclusive urban futures.

The proposed theme issue aims to catalyse meaningful interdisciplinary dialogue and collaboration among scholars in various quantitative scientific fields with potential relevance for practitioners and policymakers. This spans across fields such as Complexity and Data Science, Artificial Intelligence, Computational Social Science, traffic modeling and urban planning. The theme issue endeavours to highlight specific, emergent innovations at the nexus of digital technology and societal transformation, offering a platform for showcasing actionable research and its real-world implications.

The theme issue also aims to inform evidence-based policy discussions. As digital technology becomes ever more embedded in our societies, it is crucial for policymakers to understand the opportunities, risks and ethical implications involved. By providing insights into subjects such as digital democracy, privacy and security in the digital age, the digital divide, as well as value-sensitive technology design, this theme issue hopes to inform and guide policy decisions in a variety of contexts, both nationally and internationally.

The broader societal implications of the topics addressed in the theme issue mean that it is also likely to attract public interest. In a world where individuals increasingly interact with digital technologies in their daily lives, understanding the impact of these technologies, and the potential for using them for a more sustainable future, is of great relevance. Therefore, this theme issue can contribute to raising public awareness and promoting informed discussions about the digital revolution and its societal implications.

The objective is to move beyond high-level discussions and to discuss the specifics, providing readers with a clearer understanding of the potential impacts, challenges and opportunities presented by digital governance and participatory cities, thereby making a valuable contribution to both the academic community and wider societal discussions.

## 4. Physics and beyond—exploring new paths

We would also like to stress that all social structures are now becoming socio-technical/techno-social systems. As a result of this, it is not enough to advance technological innovation. In socio-technical systems, technology will change social interactions and outcomes, and social (inter)actions, in turn, will change the outcomes of technological interference. In other words, the areas of Smart Cities and Urban Analytics on the one hand and people-centred governance systems such as democracies on the other hand should not be studied in separation anymore. They are becoming one integrated complex dynamical system, which we need to understand better, if technology should serve us well.

Our theme issue focuses exactly on this integration of both subjects, and that is where the main innovation is to be found. In fact, we hope that this integrated, systemic view will mark a milestone in science and the way technology is being used in the future.

Driven by scientific knowledge gaps, which had to be urgently filled, we have seen the integration of scientific, social, computational, engineering sciences already for some time. This was marked by the emergence of methodology-driven fields such as Socio-, Econo- and Traffic Physics [39], which was followed by the even bigger field of Computational Social Science [40–42]. This was largely fuelled by Data and Computer Science, and by methods of investigation inspired by the natural sciences but, importantly, is now integrated with knowledge from the social sciences.

In the meantime, socially and physics-inspired methods have guided new ways of how to design systems that are better than those purely based on data-driven optimization attempts [11], and they have great potential to inform us how to run complex dynamical systems in the future. The underlying knowledge comes largely from natural science, particularly complexity science, and the future impact is expected to be high.

We would like to end on the note that, already in the days of Isaac Newton, the spectrum of publications in the journal *Philosophical Transactions A*—often called the oldest scientific journal in the English-speaking world—was reaching beyond pure physics. Hence, our theme issue is trying—in all due modesty—to reconnect with the good old tradition of this honourable journal of the ‘Royal Society’.

## Data Availability

This article has no additional data.

## Footnotes

^1^
More information about the workshop is available at https://www.participatorycities.net. The talks given during the workshop can be accessed via this YouTube playlist: https://www.youtube.com/watch?v=tZvhw2_tmEQ&list= PLk7tDwnvsZsQEqiErG_sLuOiq6mHSbdpK.

^2^
https://web.archive.org/web/20230820152814/https://www.smart-city-dialog.de/wp-content/ uploads/2019/12/smart-city-charta-langfassung.pdf (p. 43).

^3^
See the Special Issue on 'Ethics of Smart Cities and Smart Societies' in the journal on *Ethics and Information Technology*. Accessible here https://link.springer.com/collections/hefadbghfh.
